# Peer Review of “Perception and Impact of White Spot Lesions in Young People Undergoing Orthodontic Treatment and Their Guardians: Protocol for a Mixed Methods Study”

**DOI:** 10.2196/80143

**Published:** 2025-09-12

**Authors:** Jonathan Shaw

**Affiliations:** 1 School of Medicine, California University of Science and Medicine, Colton, CA, United States

**Keywords:** orthodontics, white spot lesions, fixed appliances, dentistry

*This is a peer-review report for “Perception and Impact of White Spot Lesions in Young People Undergoing Orthodontic Treatment and Their Guardians: Protocol for a Mixed Methods Study.”*

## Round 1 Review

### General Comments

 This paper [1] appears to me to be well-written and adequately cited. I believe that this paper will contribute to the literature once the study commences and the data are collected and analyzed. However, I do have some questions/concerns regarding the study design and potential data analysis that I have included in my comments below. I would like the authors of this paper to review these comments/recommendations and to either implement them as they see fit or justify why they believe they do not need to.

### Specific Comments

#### Major Comments

1. Line 170, you state that this study is purely descriptive, so a power analysis is not required. How will you control for confounding variables such as cultural beliefs which may be over- or underrepresented in your participant pool? Additionally, how will you ensure that your participant demographics allow for the generalization of this paper’s findings to patient populations outside of Liverpool?

2. Line 203, you mention that sampling will be based on age, gender, ethnicity, etc. However, Table 2 does not mention ethnicity. Could you edit Table 2 to mention ethnicity or edit Line 203 to remove ethnicity. I would recommend editing the table because I believe the participant demographics to be important, especially since different cultures may approach esthetics and health beliefs differently. This concern regarding culture connects with major comment 1.

#### Minor Comments

3. Line 129, “Sponsorship will be sort from...,” please change to “Sponsorship will be sought from…”

4. Line 167, you state that a sample size of 200 respondents is sufficient for Part 1 of the study. Could you justify this estimate in a more thorough way other than stating that it is a “pragmatic estimation?”

5. Line 173, you state that participants will be contacted on the same day as their orthodontic appointment. Will this be before or after the appointment? Will participants be compensated for their time? How will you ensure that participants’ rights are respected and that they do not feel pressured into participating?

6. Line 427, “or childs name?” please change to “or child’s name?”
